# Residual Intravenous Contrast Mimicking a Ureteral Stent on Noncontrast Computed Tomography in a Patient With Ureterolithiasis

**DOI:** 10.7759/cureus.101749

**Published:** 2026-01-17

**Authors:** Brian R Beyer, Ali Haq

**Affiliations:** 1 Medicine, Valley Health System Graduate Medical Education (GME) Consortium, Las Vegas, USA; 2 Internal Medicine, Valley Health System Graduate Medical Education (GME) Consortium, Las Vegas, USA

**Keywords:** computed tomography, radiology, residual contrast, ureteral stent, urolithiasis

## Abstract

This case describes a 53-year-old male patient presenting with right-sided abdominal pain and acute kidney injury (AKI) who retained intravenous (IV) contrast within the ureter from a previous image 24 hours prior at a different hospital that was initially mistaken for a calcified ureteral stent after presenting to a different hospital without the patient initially disclosing his previous hospital stay. This created diagnostic uncertainty and prompted subspecialty consultations for a device the patient had never received. The patient subsequently passed a ureteral calculus with resolution of symptoms and improvement in renal function. This case highlights the importance of recognizing residual IV contrast as a potential mimic of foreign bodies on computed tomography (CT), particularly in patients with recent contrast-enhanced studies.

## Introduction

Noncontrast enhanced computed tomography (CT) is the gold standard for evaluating ureterolithiasis. However, radiographic mimics may complicate interpretation and clinical decision-making, like this case. Residual intravenous (IV) contrast layering within the ureter has been described as a diagnostic pitfall, occasionally resembling calcifications or indwelling ureteral stents [1-3]. IV contrast may persist within the ureter because delayed excretion occurs when urinary flow is obstructed or renal function is impaired, allowing contrast material to pool and layer rather than clear normally. Recognition of this phenomenon is essential to avoid unnecessary interventions such as ureteroscopy with stent removal. This procedure carries risks such as anesthesia complications, ureteral injury or perforation, infection, bleeding. We present a case of ureterolithiasis in which retained contrast was initially mistaken for a calcified ureteral stent. 

## Case presentation

A 53-year-old male flight attendant with no significant past medical history presented to the emergency department in Las Vegas with three days of right-sided abdominal pain radiating to the groin. Symptoms began during a flight from Japan to California. He reported several episodes of vomiting on the flight, which subsequently resolved. He denied fever, chills, and endorsed dysuria and pain radiating to his groin. 

On arrival, the patient was hemodynamically stable, afebrile, and in mild distress. Physical examination revealed right lower quadrant and flank tenderness without guarding or rebounding. Initial laboratory results revealed an elevated creatinine with no baseline level for reference, normal blood urea nitrogen, leukocytosis, lactic acid level, and hypokalemia (Table 1). Urinalysis demonstrated blood, trace ketones, and rare mucous, but no leukocyte esterase or nitrites as shown in Tables 2-3. Blood and urine cultures obtained were negative.

**Table 1:** Laboratory values

Lab	Patient values	Reference values
Creatinine	2.24 mg/dL	0.7-1.30 mg/dL
Blood urea nitrogen	17 mg/dL	7-18 mg/dL
White blood cells	12.93 × 10³/µL	3.18-12.74 × 10³/µL
Potassium	3.3 mmol/L	3.5-5.1 mmol/L
Lactic acid	0.8 mmol/L	0.5-2.2 mmol/L

**Table 2:** Macroscopic urinalysis

Macroscopic urinalysis	Patient values	Reference values
Color	Yellow	Yellow
Ketones	Trace	Negative
Blood	3+	Negative
Nitrite	Negative	Negative
Leukocyte esterase	2+	Negative

**Table 3:** Microscopic urinalysis HPF: high power field

Microscopic urinalysis	Patient values	Reference values
White blood cells	11/HPF	0-5/HPF
Red blood cells	6/HPF	0-2/HPF
Bacteria	1+/HPF	none
Squamous epithelium	None	0-5/HPF
Mucous	Rare	None

**Figure 1:**
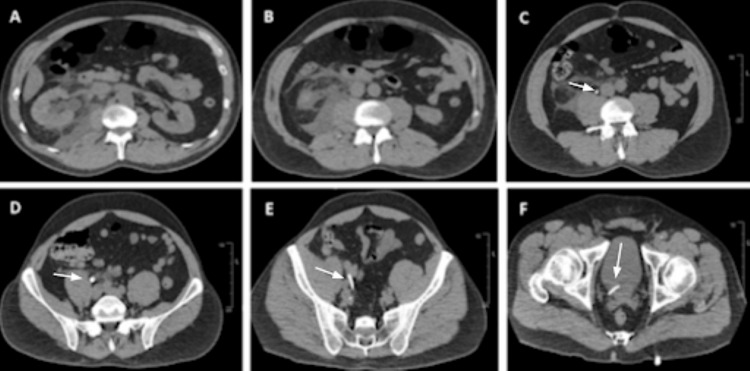
Noncontrast enhanced CT imaging of the abdomen and pelvis demonstrating right hydroureteronephrosis (A,B) with a linear high-density structure in the distal ureter extending into the bladder concerning for ureteral stent (C-F)

Noncontrast enhanced CT imaging of the abdomen and pelvis demonstrated right hydroureteronephrosis with a linear high-density structure in the distal ureter, initially concerning for a migrated or calcified ureteral stent (Figure 1). The linear high-density structure measured 244 Hounsfield units (HU). The patient denied any history of ureteral stent placement. Urology and nephrology were consulted to help evaluate the calcified stent. The patient was admitted for pain control, renal function monitoring, and management of acute kidney injury (AKI). He was treated with IV lactated Ringer's fluid, tamsulosin 0.4 mg orally daily, and analgesics, including morphine 2-4 mg IV every four hours as needed and hydrocodone-acetaminophen 10-325 mg orally every four hours as needed until he spontaneously passed the stone later during his hospital stay. Empiric ceftriaxone 1 g IV daily was initiated due to leukocytosis and flank pain.

Renal ultrasound revealed mild right hydronephrosis. During hospitalization, the patient reported spontaneous passage of a stone and rapid improvement in symptoms. The stone was visualized via a urinary strain. His creatinine improved from 2.24 mg/dL on admission to 0.93 mg/dL by discharge. Repeat noncontrast CT demonstrated persistent but improving hydronephrosis with no evidence of a stent or retained foreign body. The patient was discharged in stable condition, tolerating diet, afebrile, and pain-free. 

Upon further review, it was noted that the patient had undergone contrast-enhanced CT imaging in San Francisco one day prior to presentation of the current visit. An addendum to the CT report clarified that the hyperdense structure likely represented retained IV contrast in the ureter at the level of the ureterovesical junction. 

## Discussion

This case illustrates an important diagnostic complication in the evaluation of suspected ureterolithiasis. Residual IV contrast may persist within the urinary tract for hours following administration, particularly in the setting of obstruction, and can closely mimic a linear ureteral stent on CT. In this case, the finding raised concern for a migrated or calcified stent, prompting subspecialty consultation. Only after correlation with recent imaging was the true etiology confirmed. 

The patient’s AKI was likely multifactorial, driven by both obstructive uropathy and recent contrast exposure. Obstructive uropathy-related AKI typically results from impaired urinary drainage, causing increased intratubular pressure and creatinine rise, which often improves promptly after decompression. In contrast, contrast-associated AKI generally presents within 24-48 hours of exposure due to renal vasoconstriction and tubular toxicity, and recovery may be more gradual. In this case, renal function normalized, with a creatinine of 0.93 mg/dL on discharge. Considering the patient had conservative management with IV fluids, analgesics, and empiric antibiotics that were discontinued once culture results were negative, supports obstructive AKI as the dominant contributor to the renal dysfunction of the patient.

With presentations such as this patient, forgotten ureteral stent syndrome is at the top of the differential [4]. This phenomenon occurs when a patient has a previous history of stent placement left beyond their intended duration. The stent subsequently becomes calcified and leads to complications that are similar to other types of obstructive uropathy. 

To differentiate calcified ureteral stents, urinary calculi, and retained contrast, quantitative assessment of radiologic attenuation using HU can be helpful. On noncontrast CT, true urinary calculi typically demonstrate focal high attenuation, commonly exceeding 300 HU and often greater than 1000 HU for calcium-based stones. In contrast, metallic or polymer ureteral stents exhibit markedly higher attenuation, with reported in vitro values ranging from approximately 1600 to 2600 HU. Retained iodinated contrast demonstrates intermediate to variably high attenuation depending on concentration and timing and typically conforms to the tubular anatomy of the ureter rather than appearing as discrete, rounded calcific foci. The patient's retained contrast had 244 HU, which is more consistent with contrast rather than a stone. Evaluation using bone-window settings may further aid in distinguishing ureteral stents, which remain hyperdense, from urinary calculi [5,6].

This case emphasizes the importance of obtaining a thorough clinical history, including prior imaging, when interpreting CT findings. Misinterpretation of retained contrast as a ureteral stent led to an initial recommendation for urology to perform ureteroscopy with presumed stent removal. This procedure carries risks such as anesthesia complications, ureteral injury or perforation, infection, and bleeding. Recognizing that no stent had been placed avoided an unnecessary and potentially harmful surgery.

## Conclusions

Retained IV contrast within the ureter is an under recognized mimic of indwelling devices such as ureteral stents on noncontrast CT imaging especially in patients with recent contrast exposure in the setting of ureteral obstruction. Awareness of this phenomenon is essential to avoid diagnostic errors and unnecessary interventions such as ureteroscopy with stent removal. Careful review of prior imaging and clinical correlation can help distinguish true foreign bodies from contrast-related artifacts. 
